# Cisplatin-Induced Ototoxicity in Rats Is Driven by RIP3-Dependent Necroptosis

**DOI:** 10.3390/cells8050409

**Published:** 2019-05-02

**Authors:** Mi-Jin Choi, Hyunsook Kang, Yun Yeong Lee, Oak-Sung Choo, Jeong Hun Jang, Sung-Hee Park, Jong-Seok Moon, Seong Jun Choi, Yun-Hoon Choung

**Affiliations:** 1 Department of Otolaryngology, Ajou University School of Medicine, Suwon 16499, Korea; cmjpink@naver.com (M.-J.C.); seven260@naver.com (Y.Y.L.); oaksung@ajou.ac.kr (O.-S.C.); jhj@ajou.ac.kr (J.H.J.); soyclara99@naver.com (S.-H.P.); 2 Bk21 Plus Research Center for Biomedical Sciences, Ajou University Graduate School of Medicine, Suwon 16499, Korea; 3 Department of Otolaryngology-Head and Neck Surgery, Cheonan Hospital, Soonchunhyang University College of Medicine, Cheonan 31151, Korea; ssook4311@hanmail.net; 4 Soonchunhyang Institute of Medi-bio Science, Soonchunhyang University, Cheonan 31151, Korea; jongseok81@sch.ac.kr

**Keywords:** necroptosis, cisplatin, ototoxicity, organ of Corti, spiral ganglion neuron

## Abstract

Cisplatin-induced early-onset ototoxicity is linked to hearing loss. The mechanism by which cisplatin causes ototoxicity remains unclear. The purpose of this study was to identify the involvement of receptor-interacting protein kinase (RIP)3-dependent necroptosis in cisplatin-induced ototoxicity in vitro and in vivo. Sprague–Dawley rats (SD, 8 week) were treated via intraperitoneal (i.p.) injection with cisplatin (16 mg/kg for 1 day), and their hearing thresholds were measured by the auditory brainstem response (ABR) method. Hematoxylin and eosin (H & E) staining, immunohistochemistry, and western blots were performed to determine the effect of cisplatin-induced ototoxicity on cochlear morphology. Inhibitor experiments with necrostatin 1 (Nec-1) and Z-VAD were also performed in HEI-OC1 cell line. H&E stains revealed that the necroptotic changes were increased in the organ of Corti (OC) and spiral ganglion neurons (SGNs). Moreover, immunohistochemistry and western blot analysis showed that cisplatin treatment increased the protein levels of RIP3 in both OCs and SGNs. The treatment of Nec-1, a selective RIP1 inhibitor, resulted in markedly suppression of cisplatin-induced cell death in HEI-OC1 cells, whereas Z-VAD treatment did not change the cisplatin-induced cell death. Our results suggest that RIP3-dependent necroptosis was substantial in cisplatin-induced ototoxicity; inner cochlear regions, the OCs, and SGNs were especially sensitive to necroptosis.

## 1. Introduction

Necroptosis is defined as a programmed form of necrosis and is executed by receptor-interacting protein kinase1 (RIP1) and RIP3. Inhibition of caspase activity is necessary for necroptosis to occur and can be identified and characterized by the following: cellular rounding, an increase in cytosolic calcium ions, formation of reactive oxygen species (ROS), depletion of adenosine triphosphate (ATP), intracellular acidification, and, ultimately, cellular swelling followed by cell membrane rupture, leading to release of damage-associated molecular patterns (DAMPs) [1,2]. The tumor necrosis factor alpha (TNFα) signal pathway has been studied for its influence in necroptosis; TNFα binds to tumor necrosis factor receptor (TNFR)1, leading to its internalization and subsequent formation of death-inducing signaling complex (DISC), known as complex II. In complex II, RIP1 and RIP3 are inactivated through their proteolytic cleavage by caspase-8. However, in the absence of caspase-8, the complex II signaling cascade leads to necroptosis [2]. Necroptosis is involved in various diseases, including stroke [3] and myocardial infarction [4], and in the process of ischemia–reperfusion (IR) injury [5,6]. Ruhl et al. demonstrated that two types of programmed cell death, apoptosis and necroptosis, contribute to aminoglycoside and cisplatin ototoxicity [7]. However, Sheth et al. insisted that a low concentration of cisplatin promotes apoptosis, whereas high doses were associated with other mechanisms of cell death, such as necrosis [8]. 

Cisplatin (cis-diamine-dichloroplatinum II, CDDP) is a well-known anticancer drug. Cisplatin primarily causes cell death by intercalating DNA, leading to a major tumoritoxic effect [9]. However, the risk of ototoxic and nephrotoxic side-effects commonly hinders the use of higher doses that could otherwise maximize its antineoplastic effects [10]. The incidence of cisplatin-induced hearing loss in children ranges from 22% to 77% [11]. Cisplatin’s ototoxicity usually manifests in a bilateral, progressive, and usually irreversible sensorineural hearing loss; cisplatin primarily damages outer hair cells in the basal turn of the cochleae and spiral ganglion neurons (SGNs). Higher doses of cisplatin are associated with additional damage to inner hair cells, supporting cells, and stria vascularis [12,13]. Therefore, an understanding of its toxicity mechanism(s) is crucial in minimizing such side-effects. 

To date, the mechanism of cisplatin-induced ototoxicity has been widely researched. Previous studies on cisplatin-induced ototoxicity have shown that accumulation of ROS leads to oxidative stress and subsequent intracellular reactions, eventually leading to cell-death [14,15]. ROS causes lipid peroxidation, which, in turn, leads to stimulation of Bax (BCl2-associated X protein) in the cytosol. Bax subsequently activates caspases 3 and -9 within the damaged outer hair cells (OHCs), leading to apoptosis [16,17]. Many cisplatin-protective agents have been evaluated: sodium thiosulfate [18], glutathione (GSH) [19], GSH ester [20], vitamin C [21], and sodium salicylate [22]. However, to date, there are no methods that can completely prevent the loss of hair cells or the dysfunction of auditory nerves in cases of cisplatin-induced ototoxicity. 

In this study, we investigated the involvement of RIP3-mediated necroptosis in cisplatin-induced ototoxicity. 

## 2. Materials and Methods

### 2.1. Experimental Animal Model

**Figure 1:**
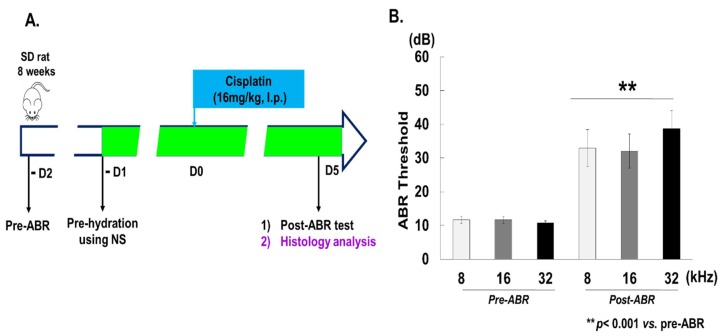
Cisplatin induces hearing loss in rats. (**A**) Schematic of the in vivo experimental procedures using Sprague–Dawley (SD) rats. ABR thresholds were measured at 8, 16, and 32 kHz 2 days prior (pre-ABR) and 5 days after (post-ABR) intraperitoneal application of cisplatin (16 mg/kg) in SD rats. (**B**) The threshold shifts (difference between pre- and post-ABR) are summarized. After the cisplatin treatment marked hearing loss were detected, with ABR thresholds increasing to 34.5 ± 16.1 dB, 40.8 ± 16.7 dB, and 43.7 ± 18.2 dB at 8, 16, and 32 kHz, respectively. NS, normal saline; dB, decibel; ABR, auditory brainstem response.

Male Sprague–Dawley rats (SD, 8 weeks) were purchased from DBL Co. (Eumseong, Korea). The rats were housed under controlled conditions with a 12-h light/dark cycle and had free access to water and food. To induce ototoxicity in the animal model, rats were treated with cisplatin (Sigma-Aldrich, St. Louis, MO, USA) at 16 mg/kg for 1 day via intraperitoneal (i.p.) injection. Prior to ototoxic injury, rats’ auditory responses were evaluated using the auditory brainstem response (ABR) method (Figure 1A). Tests were carried out after the last gentamicin (GM) and cisplatin treatments, at 2 weeks and 5 days, respectively. Following this, rats were euthanized, and the skull was dissected to obtain the cochlea for histological analysis. Animal care and studies were conducted ethically in accordance with standard protocols and approved by Institutional Animal Care and Use Committee of Ajou University Medical Center (IACUC-AUMC). 

### 2.2. Auditory Brainstem Response (ABR)

All animals were anesthetized using a mixture of 50 mg/kg Zoletil 50 (Virbac Laboratoires, Carros, France) and 4 mg/kg Rompun 2% (Bayer Korea, Ansan, Korea). ABR measurements were conducted in a sound-proof chamber using the Tucker Davis Technology (TDT) System III hardware and Biosig 32 software (Gainesville, FL, USA). For hearing threshold evaluation, needle electrodes were inserted subcutaneously at the vertex (active), under the pinna of the left ear (reference), and under the right ear (ground). ABRs were measured at frequencies of 8, 16, and 32 kHz with tone-burst stimuli reducing levels in the range of 10–90 dB, with 5dB intervals, to determine the lowest intensity level. Each measurement point was recorded and averaged 1000 times. Body temperature was monitored and maintained at 37.5 °C using a heating pad. ABR waveforms were monitored in an electrically shielded booth. The auditory threshold was defined as follows: the minimum intensity signal from stimuli that evoked waveforms with peak-to-peak voltage more than two standard deviations (SD) of the background activity (Cediel et al., 2006; Garcia-Pino et al., 2010). 

### 2.3. Histology and Immunohistochemistry

To explore both histology and immunohistochemistry, 6-μm-thick paraffin-embedded cochlear sections were used. The cochlear sections were first dewaxed using xylene, followed by rehydration through a series of graded ethanol washes, and finally subjected to histological analysis using hematoxylin and eosin (H & E) staining. For immunohistochemistry, antigen retrieval was carried out by placing slides in 10 mM sodium citrate buffer (pH 6.0) and boiling (95–98 °C) samples in a water bath for 30 min, followed by cooling at room temperature (r.t) for 30 min. Cochlear sections were then subjected to endogenous peroxidase blockage using 3% hydrogen peroxide (Sigentamicina, MO, USA) for 1 h. The sections were then incubated in blocking/permeabilization solution (3% Bovine Serum Albumin (BSA, GenDEPOT Inc., Barker, TX, USA) and 0.05% Triton X-100 in 0.1 M phosphate buffered saline (PBS)) for 1 h at r.t. Subsequently, specific primary antibodies were incubated overnight at 4 °C. Cochlear sections were washed (3× solution containing 1% BSA, 0.025% Triton X-100 in 0.1 M PBS) and then incubated with peroxidase conjugated secondary antibodies for 1 h at r.t. The sections were then washed three times with washing buffer, the sections were incubated in immunological complexes, and visualization was carried out by the addition of the 3,3–diaminobenzidine (DAB) substrate over 10 min (Abcam, CA, USA; ab64238). Sections were counterstained with hematoxylin. The negative control for the immunohistochemical procedures involved the substitution of the primary antibody with non-immune serum. Images of the sections were captured using bright field microscopy (Olympus, Tokyo, Japan).

### 2.4. Chromogenic Intensity Quantitation 

Immunohistochemistry quantifications were based on pixel intensity/area and were measured by Image J (version 1.52h; NIH). The OCs, SGNs, and lateral wall at the mid-cochlear turn, were selected as regions of interest (ROIs) for immunolabeling. Using the freehand selection tool, we selected the DAB-stained ROIs and calculated the pixel intensity/area. For intensity measurements, the mean gray value was determined by converting the RGB pixels in the image to grayscale/brightness values. The mean gray value represents the sum of the gray values of all pixels in the selection divided by the total number of pixels. The lower the pixel value, the higher the intensity. Each test group contained one cochlea per mouse (n = 3). The mean gray values and areas of the ROIs were averaged for three independent sections and presented as relative chromogenic intensity compared to the control group. 

### 2.5. Western Blot Analysis

Cochlear were dissected on ice and homogenized using a Dounce Homogenizer in radioimmunoprecipitation assay buffer (RIPA) (50 mM Tris (pH 7.5), 150 mM NaCl, 1% Nonidet P 40, 0.5% sodium deoxycholate, and 0.1% sodium dodecyl sulfate (SDS)) supplemented with 1X Protease Inhibitor Cocktail (P8340, Sigma, St. Louis, MO, USA); samples subjected to 10 stroke intervals for a total of three replicates, followed by sonication on ice. After the solubilization of proteins was complete, the concentration was measured using the Bradford blue assay (Abs 595 nm) with the Bio-Rad Protein Assay Kit (Bio-Rad, Hercules, CA, USA). Protein samples (40 µg) were loaded onto gels for electrophoresis. Proteins were transferred onto polyvinylidene difluoride (PVDF) membranes, followed by subsequent blockage with 5% non-fat dry milk in PBS with Tween-20 (PBST) buffer (137 mM NaCl, 2.7 mM KCl, 10 mM Na_2_HPO_4_, 2 mM KH_2_PO_4_) at r.t for 1 h. Membranes were incubated overnight at 4 °C with the following primary antibodies: Bax (D3R2M): #14796 (Cell Signaling Technology, Danvers, MA, USA), cleaved caspase-3 (Asp175): #9661 (Cell Signaling Technology), RIP (D94C12): #3493 (Cell Signaling Technology), RIP3: ADI-905-242 (Enzo Biochem, New York, NY, USA), and β-Actin (8H10D10): #3700 (Cell Signaling Technology). After three washes with PBST, the membranes were further incubated with horseradish peroxidase (HRP)-conjugated secondary antibodies for 1 h at r.t. Finally, the membranes were washed a further three times with PBST and then detected using enhanced chemiluminescence (ECL).

### 2.6. In Vitro Experiments 

House Ear Institute-Organ of Corti 1 (HEI-OC1) cells were cultured under non-permissive conditions (37 °C, 5% CO_2_) in high-glucose Dulbecco’s modified Eagle’s medium (Gibco, Grand Island, NY, USA) containing 10% fetal bovine serum (Gibco) without antibiotics. HEI-OC1 cells were seeded at 5 × 10^3^/cm^2^ in 96-well plates in culture medium for 24  h. Cells were pre-treated with Nec-1(10 µM) (Calbiochem, San Diego, CA, USA) or Z-VAD (100 nM) (Sigma) for 1 h before cisplatin (10 µM) was added and incubated for 24 h. Next, 10% *v*/*v* WST-1 (Cayman Chemical Company, city, state abbrev if USA/Canada, country) was added to each well and incubated for additional 2 h according to the manufacturer’s protocol. The absorbance at 450 nm was measured. The samples were assayed in at least triplicate using the iMark Microplate Reader (Bio-Rad).

### 2.7. Statistical Analysis

Data are presented as means ± S.D. or standard error of the mean (S.E.M) when n = at least two independent experiments. The statistical significance of the quantitative results was analyzed by one way analysis of variance (ANOVA) for comparisons between multiple groups. Using Statistical Package for the Social Sciences (SPSS) software (version 14.0, IBM Corporation, Armonk, NY, USA), we performed further analysis in the form of the post hoc Tukey’s honestly significant difference test (HSD). A probability value of less than 0.05 was considered statistically significant.

## 3. Results

### 3.1. Cisplatin Induces Hearing Loss 

For the ototoxicity experiments, SD rats (n = 10) were treated with cisplatin (16 mg/kg) for 1 day [23]. The hearing thresholds of ABR were measured using 8, 16, and 32 kHz based at the mid-basal turn of the cochlea. This ROI was chosen as the hair cell damage caused by cisplatin was most substantial at the mid-basal turn. The mean hearing thresholds at 8, 16, and 32 kHz before cisplatin treatment were 12.5 ± 4.62 dB, 10.6 ± 1.76 dB, and 12.5 ± 4.62 dB, respectively (Figure 1B). Five days after the cisplatin treatment, marked hearing loss was detected, with thresholds increasing to 34.5 ± 16.1 dB, 40.8 ± 16.7 dB, and 43.7 ± 18.2 dB at 8, 16, and 32 kHz, respectively (Figure 1B).

### 3.2. Cisplatin Induces OC and SGNs Injuries

**Figure 2:**
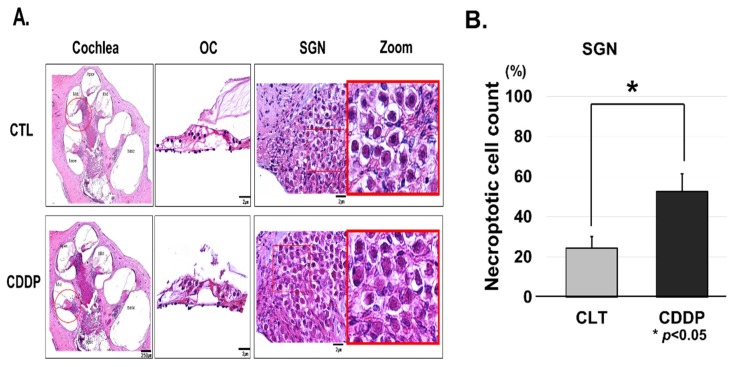
Cisplatin induces OC and SGN injury in vivo. (**A**) Hematoxylin and eosin stain. Necroptotic changes were pronounced in the OCs and SGNs in cisplatin-induced ototoxicity compared to the control. (**B**) Quantitative analysis of necroptotic cell count in SGN region. We counted a number of necroptotic cell in SNG regions. The numbers were counted as to how many of the hundred SGN cells were necroptotic and statistical analysis was performed using ANOVA. Necroptotic cells in CDDP group were significantly counted than those in control. OC, organ of Corti; SGN, spiral ganglion neuron; CTL, control; CDDP, cisplatin; ANOVA, analysis of variance.

Histological analysis using H&E staining was performed to determine whether these changes in hearing ability were associated with morphologic abnormalities of cochlea. Because many studies have reported that the ototoxic drugs affect hair cells, lateral wall tissues (spiral ligament and stria vascularis), and SGNs within the cochlea, we focused on these three regions [24,25,26]. Cisplatin-treated models showed that morphologic changes were pronounced in the OCs and SGNs when compared to the control model (Figure 2). This was particularly noticeable for SGNs, where there were cell death features such as necroptotic morphology (Figure 2A). Furthermore, for quantitative analysis we counted a number of necroptotic cell in SGN regions five times in total. The numbers were counted as to how many of the hundred SGN cells were necroptotic and statistical analysis was performed using ANOVA. Necroptotic changes in cisplatin-treated rats were significantly observed than those in control (Figure 2B). 

### 3.3. Cisplatin Increases RIP3 Expression in OC and SGN

It is generally accepted that ototoxic drugs induce cellular damage and death in auditory cells through the activation of apoptosis, necroptosis, and/or autophagy, resulting in hearing loss. Recent studies have shown that apoptotic/autophagic signaling pathways play important roles in aminoglycoside-induced cell death [27,28]. In contrast to aminoglycosides, cisplatin has been proposed to cause cellular toxicity through other distinct mechanisms [8].

**Figure 3:**
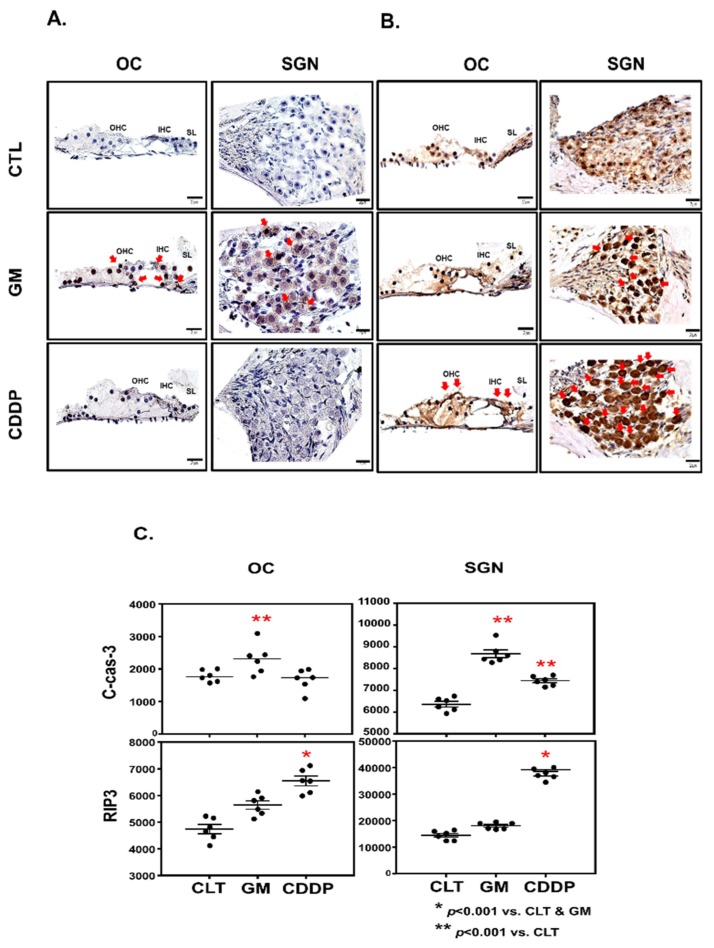
Cisplatin increases receptor-interacting protein kinase3 (RIP3) expression in the OCs and SGNs. (**A**) Immunhistochemistry of cleaved caspase-3. Cleaved caspase-3 expression of rats treated with GM was highly observed throughout SGNs, as well as OHCs and IHCs in the OC. (**B**) Immunhistochemistry of RIP3. RIP3 expression of cisplatin-treated rats was highly observed throughout SGNs, as well as OHCs and IHCs in the OC. (**C**) Chromogenic intensity quantitation of immunohistochemistry. RIP3 showed higher relative intensity in OC and SG in the cisplatin group than the control and GM groups. In contrast, the GM group showed the significant change of C-cas-3 expression in OCs and SGNs compared to the control and cisplatin groups. OC, organ of Corti; SGNs, spiral ganglion neurons; OHC, outer hair cell; IHC, inner hair cell; SL, spiral limbus; CTL, control; CDDP, cisplatin; GM, gentamicin; C-cas-3, cleaved caspase-3.

To investigate the localization and expression profiles of RIP3 in the cochlea after cisplatin injection, SD-rats were given intraperitoneal injections of cisplatin for 1day (16 mg/kg body weight per injection). Immunohistochemistry was performed in the mid-basal turn of cochlea. In control rats, the staining of RIP3 proteins was barely detectable except weakly stained spiral limbus regions. However, after injection with cisplatin, RIP3 staining was prominently observed throughout SGNs, as well as OHCs and IHCs in the OC (Figure 3A). To quantitatively analyze immunohistochemistry, we used the chromogenic intensity quantitation (Figure 3B). RIP3 showed higher relative intensity in OC and SG in the cisplatin group than the control and GM groups. In contrast, the GM group did not show the change of RIP3 expression in OCs and SGNs compared to the control group, but showed a significant change of cleaved caspase-3 expression in OCs and SGNs than the control and cisplatin group. It means that the signal pathway of necroptosis might be the major cell death mechanism in cisplatin-induced ototoxicity, and the regions sensitive to necroptosis might be OCs and SGs.

### 3.4. Cisplatin Promotes RIP3-Dependent Necroptosis in Cochlea

**Figure 4:**
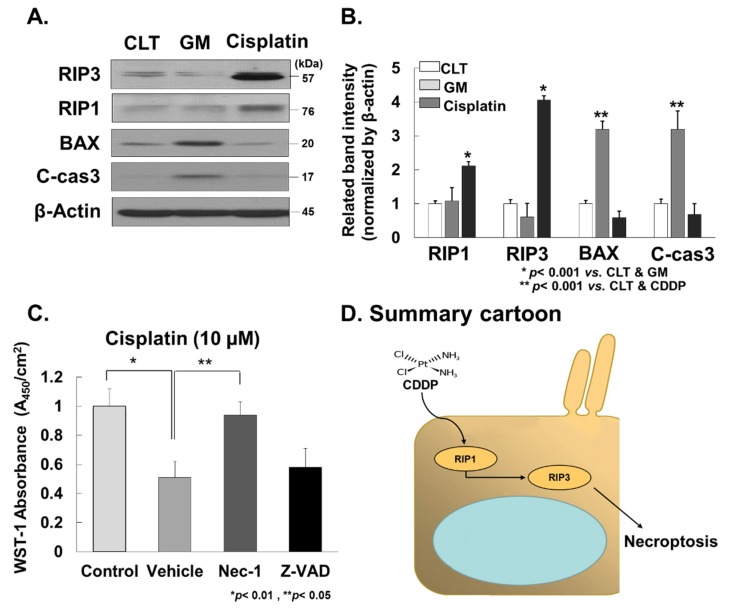
Cisplatin promotes RIP3-dependent necroptosis in cochlea. (**A**) Western blot. RIP1 and RIP3 protein expression levels were significantly elevated in cochlear tissues treated with cisplatin compared to the vehicle control. However, in GM-treated rats BAX and cleaved caspase-3 protein expression were significantly elevated in cochlear tissues compared to the vehicle control. (**B**) Densitometry analysis. (**C**) Inhibitor experiment in the HEI-OC1 cell line. HEI-OC1 cells were pre-treated with Nec-1(10 µM) or Z-VAD (100 nM) for 1 h before cisplatin (10 µM) was added. Nec-1 showed a markedly protective effect for the HEI-OC1 cell line treated with cisplatin (10 µM), but Z-VAD did not show this. (**D**) Summary cartoon. CTL, control; GM, gentamicin. C-cas-3, cleaved caspase-3; CDDP, cisplatin; Nec-1, necrostain-1.

Investigation into whether cisplatin could promote RIP3-dependent necroptosis in cochlea was carried out. We measured the expression levels of RIP1 and RIP3 in the cochlear tissues collected from rats treated with cisplatin. Notably, RIP1 and RIP3 protein expression levels were significantly elevated in cochlear tissues treated with cisplatin compared to the vehicle control (Figure 4A,B and Figure S1). In contrast, the expression levels of RIP1 and RIP3 in GM treated rats did not change relative to the vehicle control. Furthermore, we performed the inhibitor experiments with Nec-1 and Z-VAD in the HEI-OC1 cell line. Nec-1 showed markedly protective effect of HEI-OC1 cell line treated with cisplatin (10 µM), but Z-VAD did not show this behaviour. These results suggest that cisplatin promotes RIP3-dependent necroptosis in cochlear tissues during cisplatin-induced ototoxicity.

## 4. Discussion

Using immunohistochemistry and western blot analysis, we confirmed that RIP3-dependent necroptosis plays an important role in cisplatin-induced ototoxicity. In contrast, GM, which is known to have a toxic mechanism similar to that of ROS, showed less RIP3 expression than cisplatin. These results indicate that distinct developmental strategies for preventative drugs are needed to tackle the differing mechanisms of toxicity shown by cisplatin and GM. 

Necroptosis is executed by RIP1 and RIP3 when apoptosis-mediating caspases are inhibited. RIP1 and RIP3 are involved in inflammation and cell death, and mixed lineage kinase domain-like protein (MLKL) is activated by RIP3-mediated phosphorylation [29,30]. Many studies have reported that cisplatin-induced activation of caspase-3 and -9 was seen in HEI/OC1 cells [31,32]. Wang et al. also demonstrated that intra-cochlear perfusion of specific inhibitors of caspase-3 and -9 helped protect against cisplatin-induced hair cell death in animal models [17]. These studies suggest that the principle mechanism of cisplatin-induced ototoxicity is apoptosis. In contrast, our studies indicate that cisplatin-induced ototoxicity is caused by necroptosis. At pathological and biochemical levels, necroptotic-like cell death featured the following according to H & E staining: swelling of cytoplasmic organelles, rupture of plasma membrane, and release of cell contents. Additionally, according to immunohistochemistry, cisplatin-induced ototoxicity was significantly correlated with high expression levels of RIP3 in the OC and SGNs. Furthermore, western blot analysis showed cisplatin treatment increased the accumulation of RIP1 and RIP3 to a remarkable degree. Containing receptor-interacting serine/threonine-protein kinase (RIPK)1 and RIPK3, the multiple protein complex, namely the necrosome, reflected the necroptotic cell death pathway. Additionally, we performed the inhibitor experiments with the HEI-OC1 cell line treated with cisplatin. Our results showed that Nec-1, a selective RIP1 inhibitor, prevented auditory cell death more effectively than Z-VAD. Although we found that the activation of necroptosis is a major cell death pathway in cisplatin-induced ototoxicity, there is an increase of cleaved caspase-3, the active form of caspase-3, in SGNs in vivo. Since the phenotype of cell death in primary tissues in vivo might be complicated, it is difficult to exclude other cell death phenotypes such as apoptosis or necrosis. Thus, our results suggest that necroptosis is a major cell death pathway during cisplatin-induced ototoxicity. However, further research on RIP3 knock-out mice in cisplatin-induced ototoxicity is required to fully understand the role of RIP3. 

To date, a few studies have reported the role of necroptosis in various ototoxicity profiles. Zheng et al. reported that necrosis and noise-induced outer hair cell apoptosis were modulated by caspases and RIP kinases. Inhibition of either pathway resulted in a prevalence shift of outer hair cell death to the other pathway [33]. Park et al. demonstrated the protective effect of Necro X, a necrosis/necroptosis inhibitor, on GM-induced hair cell loss in neonatal cochlea cultures, suggesting that it may have therapeutic potential in the treatment of drug-induced ototoxicity [34]. However, it was suggested that Necro X showed protective effects only for hair cells, with anti-apoptotic and anti-oxidative, not anti-necroptotic, activities. Wang et al. also reported that ouabain-induced SGN injury promoted an increase in RIP3 expression but could be suppressed by application of the necroptosis inhibitor Nec-1 [35]. However, Ruhl et al. reported that the protective effect of Nec-1 was not reflected in an ex vivo experiment that employed cisplatin induced ototoxicity. It was suggested that the differences in the activity profiles of Necro X and Nec-1 were due to the kinase selectivity profiles, including the off-target inhibition of related kinases [7]. These results show that drug-induced ototoxicity is very complex; the exact mechanisms for the intracellular processing of RIP1/RIP3-cell death are still unknown, and the identity of the inner-ear target cell types that are sensitive to RIP3-mediated necroptosis remains unresolved. 

There is compelling evidence that ROS production plays an important role in cisplatin-induced ototoxicity [36]. To date, many studies also report a relationship between ROS formation and the apoptosis of hair cells [17,26]. However, the exact mechanism of ROS-induced apoptosis remains unclear. Additionally, there is currently no literature outlining the relationship between ROS formation and necroptosis in various ototoxic diseases. Many studies in other fields have reported that ROS production is necessary for necroptosis in several cell lines, such as macrophages and 1929 cells [37,38]. Wang et al. reported that AMP-activated protein kinase (AMPK) protected against myocardial IR injury caused by ROS-induced necroptosis [39]. Meng et al. also demonstrated that the inhibition of ROS suppressed RIP-mediated human kidney (HK) 2 cell necroptosis, which may be the principle mechanism of cisplatin-induced nephrotoxicity [40]. Therefore, we thought that further research into ROS-mediated necroptosis in various ototoxic diseases is required to fully understand cisplatin-induced ototoxicity. 

In conclusion, our results suggest that RIP3-dependent necroptosis was highly expressed in cisplatin induced ototoxicity, and the regions within the cochlea that were particularly susceptible to necroptosis were the OCs and SGNs. 

## Supplementary Materials

The following are available online at https://www.mdpi.com/2073-4409/8/5/409/s1, Figure S1: Western blot of whole cochlear tissue.
