# Supplementary material for: Cisplatin-Induced Ototoxicity in Rats Is Driven by RIP3-Dependent Necroptosis
Source: Cells. 2019 May 2;8(5):409. doi: 10.3390/cells8050409 (PMC6562419; doi:10.3390/cells8050409)
Supplement: Supplementary file 1 [file cells-08-00409-s001.pdf]

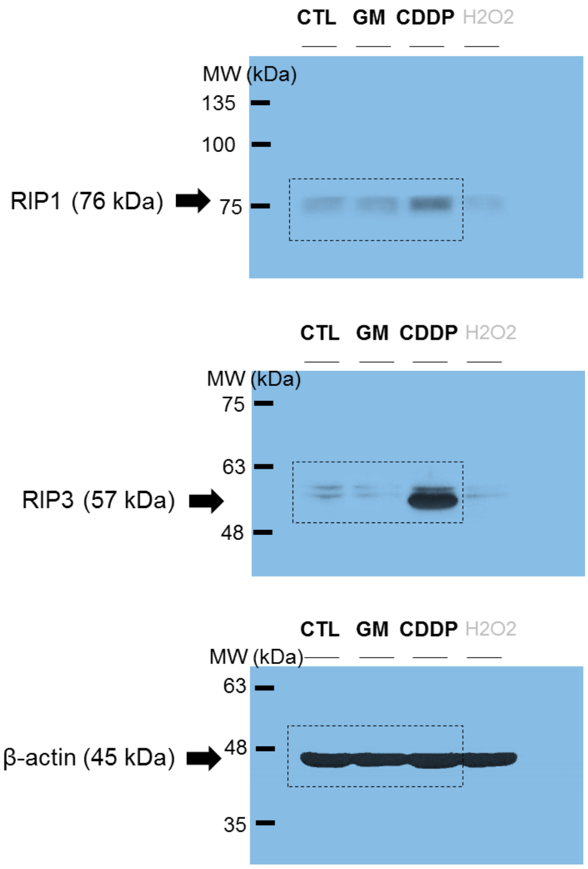

Supplementary Figure S1: Western blot of whole cochlear tissue. RIP1 and RIP3 protein expression levels were significantly elevated in cochlear tissues treated with cisplatin compared to the vehicle control and the GM group. CTL, control; GM, gentamicin; CDDP, cisplatin.
